# Bicenter validation of a risk model for the preoperative prediction of extraprostatic extension of localized prostate cancer combining clinical and multiparametric MRI parameters

**DOI:** 10.1007/s00345-024-05232-6

**Published:** 2024-09-20

**Authors:** Nicola Edith von Ostau, Analena Elisa Handke, Manuel Wiesenfarth, Peter Albers, Gerald Antoch, Joachim Noldus, Henning Reis, Cristina Cotarelo, Julia Preetz, Lale Umutlu, Marc Ingenwerth, Jan Philipp Radtke, Boris Hadaschik, Lars Schimmöller, Claudia Kesch

**Affiliations:** 1 Department of Urology, University Hospital Essen, Hufelandstraße 55, 45147 Essen, Germany; 2 Department of Urology, Ruhr-University Bochum, Marien Hospital, Herne, Germany; 3 Division of Biostatistics, German Cancer Research Center, Heidelberg, Germany; 4 Department of Urology, University Hospital Düsseldorf, Düsseldorf, Germany; 5 Medical Faculty, Department of Diagnostic and Interventional Radiology, University Düsseldorf, D-40225 Dusseldorf, Germany; 6 Division of Pathology, University Hospital Frankfurt, Frankfurt, Germany; 7 Department of Pathology, Heinrich-Heine-University Düsseldorf, Düsseldorf, Germany; 8 Department of Pathology, University Hospital Mannheim, Mannheim, Germany; 9 Division of Radiology, University Hospital Essen, Essen, Germany; 10 Department of Pathology, University Hospital Essen, Essen, Germany; 11 Division of Radiology, German Cancer Research Center, Heidelberg, Germany; 12 Department of Diagnostic, Interventional Radiology and Nuclear Medicine, Marien Hospital Herne, University Hospital of the Ruhr-University Bochum, Herne, Germany

**Keywords:** Prostate cancer, Extraprostatic extension, Multiparametric MRI, Positive surgical margins

## Abstract

**Background:**

This study aimed to validate a previously published risk model (RM) which combines clinical and multiparametric MRI (mpMRI) parameters to predict extraprostatic extension (EPE) of prostate cancer (PC) prior to radical prostatectomy (RP).

**Materials and methods:**

A previously published RM combining clinical with mpMRI parameters including European Society of Urogenital Radiology (ESUR) classification for EPE was retrospectively evaluated in a cohort of two urological university hospitals in Germany. Consecutive patients (*n* = 205, January 2015 –June 2021) with available preoperative MRI images, clinical information including PSA, prostate volume, ESUR classification for EPE, histopathological results of MRI-fusion biopsy and RP specimen were included. Validation was performed by receiver operating characteristic analysis and calibration plots. The RM‘s performance was compared to ESUR criteria.

**Results:**

Histopathological T3 stage was detected in 43% of the patients (*n* = 89); 45% at Essen and 42% at Düsseldorf. Discrimination performance between pT2 and pT3 of the RM in the entire cohort was AUC = 0.86 (AUC = 0.88 at site 1 and AUC = 0.85 at site 2). Calibration was good over the entire probability range. The discrimination performance of ESUR classification alone was comparable (AUC = 0.87).

**Conclusions:**

The RM showed good discriminative performance to predict EPE for decision-making for RP as a patient-tailored risk stratification. However, when experienced MRI reading is available, standardized MRI reading with ESUR scoring is comparable regarding information outcome. A main limitation is the potentially limited transferability to other populations because of the high prevalence of EPE in our subgroups.

## Introduction

The decision-making process of planning a radical prostatectomy (RP) and estimating a patient’s prognosis in prostate cancer (PC) is still controversial [1, 2]. The traditional approach of local staging with prostate specific antigen (PSA), biopsy International Society of Urological Pathology (ISUP) grading and clinical T-stage on digital-rectal examination (DRE), does not appear to reliably predict extraprostatic extension (EPE, cT3a) [3, 4]. The introduction of multiparametric MRI (mpMRI) of the prostate has significantly improved the prognostic value of clinical parameters [5]. This has led to better preoperative prediction of EPE.

The integration of clinical data with mpMRI findings has gained significant importance for urologists in order to better stratify those men who may undergo oncological secure nerve-sparing RP [6, 7]. There has been a considerable interest in developing refined clinical tools and multivariable risk models (RM) able to predict the probability of EPE [6–8]. There is evidence suggesting that the addition of MRI findings to clinical information increases the accuracy of diagnosis, but only few of these models have been either internally or externally validated [1, 5, 9, 10]. Therefore, there is a need of further robust validation studies.

The purpose of this study was to validate a previously published RM predicting the probability of EPE based on clinical parameters and MRI features in patients who underwent MRI prior to RP and to compare the RM to the European Society of Urogenital Radiology (ESUR) classification for EPE [11, 12].

## Materials and methods

### Study population

The study population comprised 352 consecutive patients who underwent mpMRI and subsequent radical prostatectomy (RP). The analysed data included consecutive patients from two different sites (Site 1 = University hospital Essen with *n* = 75 and Site 2 = University hospital Düsseldorf with *n* = 130), subsequent systematic and targeted biopsy and RP. 147 patients (54/129 from site 1, 93/223 from site 2) were excluded from analysis due to incomplete data. The evaluation of each cohort was approved by the local ethics committee at University Hospital Essen (19-8978-BO) and University Hospital Düsseldorf (2018-227-RetroDEuA). Patients were enrolled and registered into a retrospective database assessing RP between January 2018 and June 2021in Essen and from January 2015 until December 2017 in Düsseldorf prior to RP with personal resources being the reason for the two different time frames.

Data were retrospectively analysed. Key inclusion criteria were available information on PSA, clinical T-stage, ISUP Grade group (GG) mpMRI with Prostate Imaging Reporting and Data System (PI-RADS) and RP specimen [13]. MRI examinations were classified according to PI-RADS and PI-QUAL (for analysis all classifications were transferred to v2.1). In Essen most MRIs (74/129) were performed in external institutions. All MRIs were re-read by an expert uro-radiologist (LU). In Düsseldorf, all MRIs were read/supervised by an expert uro-radiologist (LS) and all of them were performed in the centre.

Side-specific DRE staging information was collected before biopsy by the treating urologist during routine clinical care. Both side-specific DRE and mpMRI staging information were subdivided into three subclasses. These included nonpalpable disease (T1), organ-confined localized disease (T2), EPE (T3a), seminal vesical invasion (T3b) or T4.

### Imaging

All mpMRI examinations were acquired according to international recommendations measured by PI-QUAL score at 3-Tesla scanners with high imaging quality [13, 14]. Prostate volume, PI-RADS classification, index lesion (IL) with size and capsule contact length (CCL) and the clinical T-stage (cT2a to cT3b) were assessed on mpMRI, predominantly sequence T2w, before biopsy. Prostate volume (PV), CCL, lesion diameter and lesion volume were retrospectively determined. ESUR classification for EPE includes dedicated criteria for assessing extraprostatic tumour extension, seminal vesical invasion, and involvement of the bladder neck. The read was done routinely according to PIRADSv2.1 before surgery by specialized uro-/radiologists (LS, LU). Only in case of no written report was available for the analysis the mpMRT was retrospectively re-read blinded to clinical and histopathological parameters (LS, JPR, LU) [13]. The same findings were used for both RM and ESUR.

### MRI/TRUS fusion protocol

All men underwent transperineal or transrectal targeted biopsies of MRI reported suspicious lesions and systematic biopsy. At both sites, MRI/TRUS-fusion biopsy was performed using the Invivo Uronav platform, Philips, Gainesville, FL, USA. At the Essen site, *n* = 5 TB per lesion were performed, whereas at Düsseldorf *n* = 2 TB were facilitated.

### Radical prostatectomy

188 (92%) men underwent robot-assisted radical prostatectomy (RARP) and 17 (8%) retropubic RP. Each RP was performed by or under supervision of one of four experienced surgeons, each with at least 10 year of experience, having performed > 200 RPs. The surgeon was aware of MRI results.

### Histopathology

Histopathological workup followed current guidelines and local Standard Operation Procedures (SOPs) including complete embedding of the prostate. Reporting was done under the supervision of expert urogenital pathologists following ISUP and WHO criteria. All relevant data including TNM-information and Gleason Grades were reported adherent to national guidelines and ISUP criteria. If EPE was observed, the laterality (left, right, or both hemispheres) was reported. EPE was defined as a tumor that bulges the prostate contour, with direct extension into the periprostatic (fat) tissue, in the posterolateral area or invasion of the neurovascular bundle. The distinction between focal and established EPE was reported, but not taken into consideration in this manuscript.

### Statistical analysis

Patient demographics, MRI and RP results were analysed descriptively. Detailed information on RM development, have been published previously [15]. The regression equation for the RM including clinical T-stage from DRE, ISUP grade, PSA, mpMRI information (prostate volume in ml and CCL in mm) and the ESUR score was as follows:

$$\begin{aligned}\text{log}\left(\frac{{\pi\:}_{i}}{1-{\pi\:}_{i}}\right)=\\&-0.4846\:+\:0.1933\:ESU{R}_{Score}\\&+\:1.0096\:I(clinical\_T\_stage\:=T2b/c)\:\:\\&+\:\:2.6804\:I(clinical\_T\_stage=T3/4)\\&-0.9928\:log(MRI\_Volume)\\&+\:0.493\:log\left(PSA\right)-0.0749\:I(ISUP=2)\\&+\:0.7085\:I(ISUP=3)\\&+\:1.19421\:I(ISUP=4)+\:1.1833\:I(ISUP=5)\\&+\:0.1004\:MRI\_Capsule\_contact\_length\end{aligned}$$

where $\:\text{log}\left(\frac{{\pi\:}_{i}}{1-{\pi\:}_{i}}\right)$ is the logit, $\:clinical\_T\_stage\:$is the T stage grouped into T1/2a, T2b/c and T3/4, $\:MRI\_Volume$ is the prostate volume in *ml* and $\:MRI\_Capsule\_contact\_length$ is the length the tumor is in contact with the capsule in *mm*. $\:I(clinical\_T\_stage=j)$ denotes the dummy variable which is 1 if $\:clinical\_T\_stage=j$ for j= $\:T2b/c,T3/4$ (reference category T1/2a) and 0 otherwise, similarly for I(ISUP=j) with reference category ISUP=1.

We further compared the RM above to ESUR classification only (with model development on data from [12]) and equally externally validated it on the present data set. The formula was as follows:

$$\:\text{log}\left(\frac{{\pi\:}_{i}}{1-{\pi\:}_{i}}\right)=-2.6540\:+\:0.4976\:\text{E}\text{S}\text{U}\text{R}\_\text{S}\text{c}\text{o}\text{r}\text{e}$$

**Fig. 1:**
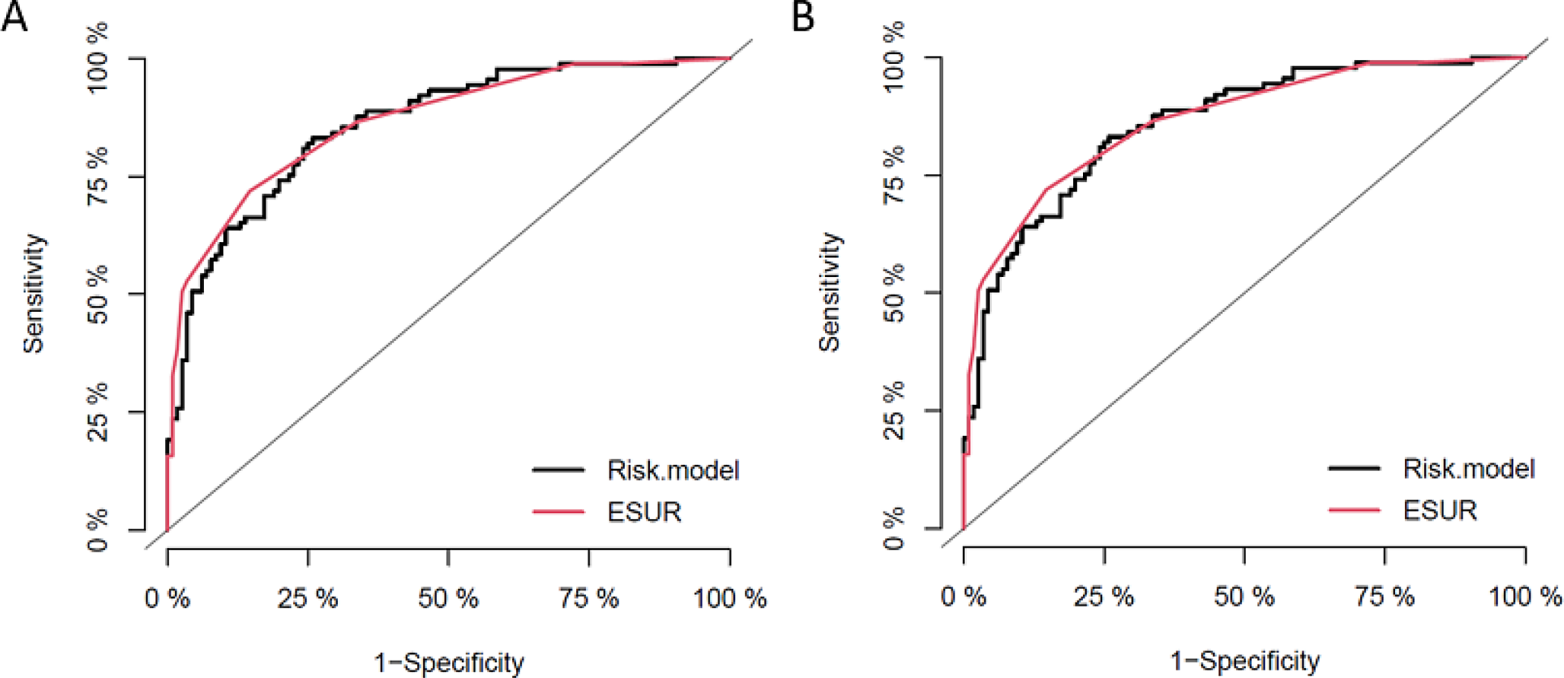
ROC curve analysis of the EPE-RM (black curve) and ESUR (red curve) for the prediction of an EPE. **A**: Site 1 (Essen); **B**: Site 2 (Düsseldorf)

Discrimination performance of the RM was assessed using Area-under-the-curve (AUC) of receiver-operating-characteristic (ROC) curve analysis and compared to ESUR classification alone (Supplemental Table 1; Fig. 1). DeLong 95% confidence intervals for AUCs are provided. Statistical differences between AUCs of prediction models were analysed using DeLong’s test for two correlated ROC curves. All tests performed were two sided, with a significance level of 5%.

The extent of over- or underestimation of predicted probabilities relative to observed probabilities of sPC was explored graphically using calibration plots for the entire cohort and for both subcohorts separately.

Statistical analyses were performed using R version 4.2.1 (R Foundation for Statistical Computing, Vienna, Austria), packages ModelGood and Calibration Curves [16–18]. Reporting followed Standards of Reporting of Diagnostic Accuracy (STARD) guidelines (S4 Table) [19].

## Results

Patient characteristics, MRI and histopathological data are given in Table 1. EPE was detected in 89 patients (43%). Stratification by centres revealed EPE in 34 men (45%) at site 1 and 55 (42%) men at site 2 (Table 2). First, we assessed the discrimination performance of the RM published by Nyarangi-Dix et al. in our data set (Fig. 1, Supplemental Table 1) [11, 13, 20]. The AUC of the RM in the entire cohort was 0.86 (95% CI: 0.81–0.91) (Supplemental Table 1). In subgroup analyses, the RM`s AUC in Site 1 was 0.88 (95% CI: 0.81–0.96) (Fig. 1A), whereas discrimination in Site 2 was 0.85 (95% CI: 0.78–0.92) (Fig. 1B).

**Table 1:** Patients’ baseline characteristics of the total cohort and in subgroups of site 1 (Essen) and site 2 (Düsseldorf) including baseline clinical parameters, biopsy and prostatectomy results

Baseline characteristics	all	Site 1	Site 2
Number	205	75	130
**Clinical parameter**			
Median Age, years (IQR)	67 (61–71)	67 (61–70)	67 (62–72)
Median iPSA-Level (IQR), ng/ml	8.9 (6.5–13.4)	8.3 (5.6–12.0)	9.0 (7.0-13.9)
Suspicious DRE findings (≥ T2), n (%)	22 (10.7)	18 (24)	4 (2.0)
Median prostate volume (IQR), ml	40 (30–57)	40 (33–60)	37 (30–50)
Median PSA density (IQR)	0.23 (0.14–0.36)	0.18 (0.12–0.27)	0.25 (0.17–0.38)
**Histopathology biopsy**			
ISUP 1, n (%)	15 (7)	3 (4)	12 (9.2)
ISUP 2, n (%)	84 (41)	33 (44)	51 (39)
ISUP 3, n (%)	38 (19)	13 (17)	25 (19)
ISUP 4, n (%)	39 (19)	14 (19)	25 (19)
ISUP 5, n (%)	29 (14)	12 (16)	17 (13)
**Histopathology RP**			
pT2a/b, n (%)	16 (7)	6 (8)	10 (8)
pT2c, n (%)	100 (49)	35 (47)	65 (50)
pT3a, n (%)	47 (23)	21 (28)	26 (20)
pT3b, n (%)	42 (20)	13 (17)	29 (22)
ISUP 1, n (%)	5 (3)	2 (2.7)	3 (2)
ISUP 2, n (%)	99 (48)	37 (49)	62 (47)
ISUP 3, n (%)	54 (26)	26 (35)	28 (22)
ISUP 4, n (%)	17 (8)	3 (4)	14 (11)
ISUP 5, n (%)	30 (15)	7 (9.3)	23 (18)
**PI-RADS**,** highest Score**			
PI-RADS III (%)	6 (3.9)	3 (4)	3 (2)
PI-RADS IV (%)	77 (37)	25 (33)	52 (40)
PI-RADS V (%)	122 (60)	47 (63)	75 (58)

*IQR* = Interquartile range, *iPSA* = Initial prostate specific antigen, *ng* = nanogram, *ml* = milliliter, *DRE* = Digital rectal examination; *ISUP* = International Society of Urological Pathology, *RP* = Radical prostatectomy

**Table 2:** Patient characteristics according to values of the risk model (predicted pT2 vs. pT3) before RP

Predicted tumor classification	pT ≤ 2c	pT ≥ 3a = EPE
Site 1, n (%)	41 (55)	34 (45)
Site 2, n (%)	75 (58)	55 (42)
Median ESUR classification for EPE (IQR)	3(2–4)	7(4–9)
Median MRI prostate volume (IQR), ml	40(30.8–54.1)	40(30–60)
Median iPSA (IQR), ng/ml	8.3(6-11.1)	9.8(7.3–16.3)
Median CCL on MRI (IQR), mm	10(6–14)	20(14–28)
Clinical T stage, cT1/2a (%)	111(61)	72(39)
Clinical T stage, cT2b/c (%)	5(39)	8(61.5)
Clinical T stage, cT3/4 (%)	0(0)	9(100)
ISUP 1 in biopsy, n (%)	13(87)	2(13)
ISUP 2 in biopsy, n (%)	58(69)	26(31)
ISUP 3 in biopsy, n (%)	23(61)	15(40)
ISUP 4 in biopsy, n (%)	18(46)	21(54)
ISUP 5 in biopsy, n (%)	4(14)	25(86)

*n* = Number, *IQR* = Interquartile range, *iPSA* = Initial prostate specific antigen, *ng* = nanogram, *ml* = milliliter, *DRE* = Digital rectal examination; *ISUP* = International Society of Urological Pathology, *RP* = Radical prostatectomy

Second, we assessed the discrimination performance of the ESUR classification for EPE prediction alone. The discrimination performance was comparable to the performance of the RM for the entire cohort (0.87, 95% CI: 0.82–0.91) (Supplemental Table 1). Comparing the AUCs for RM and ESUR alone, no significant difference could be demonstrated (*p* = 0.75). This was also the case for the subgroups: The ESUR scores` AUC at site 1 was 0.89 (95% CI: 0.81–0.96 DeLong) (Fig. 1A), and 0.86 at site 2 subgroup (95% CI: 0.80–0.92 DeLong) (Fig. 1B).

Calibration plots of the RM (Supplemental Figs. 1, 3) demonstrate that there are no untoward deviations of the predicted from the observed risk of EPE over the entire range at site 1 (Supplemental Fig. 1A) and site 2 (Supplemental Fig. 1B), with slight overestimation of EPE risk in Essen. Despite a good calibration in general, the ESUR classification for EPE alone (Supplemental Figs. 2 and 3) underestimates the observed pathological risk of EPE at higher probabilities of over 25%. However, this observation is only prevalent in the Düsseldorf cohort (Supplemental Fig. 2B) and may be induced by a limited proportion of observed / predicted EPE.

## Discussion

The previously described EPE-RM was one of the first approaches combining mpMRI and clinical parameters, including histopathological results from MRI/TRUS fusion biopsy, for a side-specific prediction of EPE in RP specimens [11]. It was now validated on bicentric data from clinical routine.

The quality of MRI for EPE prediction depends on the experience and specialisation of the radiologist, and hence may vary greatly, although standardization and implemented guidelines lead to comparability (with time) [21–23]. Unless the expertise of the radiologist is very high, the risk model may not offer an advantage over MRI findings alone [24]. However, due to the high variance in the field, the RM might offer a way to better assess the risk for EPE. Therefore, the EPE-RM integrated CCL as objectively measured parameter, histopathological data and the standardised ESUR classification as predictors of EPE to increase reproducibility and decrease reader-dependency [11].

When discussing the broad usability of the RM and ESUR, the technical limitations of MRI itself should also be considered. Microscopic EPE is hard to detect on MRI. The detection probability is higher in a 3 Tesla MRI like it was used in the original study as well as in this validation study. Studies have shown that 1.5 Tesla devices provide a slightly lower spatial resolution, which would influence the predictive ability of the risk model [25].

The main result of our analysis is that the RM performs well in two different external validation cohorts with an AUC of 0.86 in the entire cohort. Both cohorts were comparable regarding the prevalence of EPE (site 1 45%, site 2 42%). Our results further support that standardized mpMRI reading is reliable within a RM combining MRI and clinical parameters. This is in line with recent results demonstrating added benefit of MRI in combination with clinical parameters [26]. Baco et al. [27] demonstrated that the CCL on MRI can predict EPE accurately.

However, we also demonstrate a good performance of the ESUR classification alone. The ESUR classification has repeatedly been validated, is reliable for EPE prediction with AUC values up to 0.86, and may attenuate the low sensitivity of MRI [26, 28]. In the original study of Nyarangi-Dix the AUC of the ESUR classification was high with 0.81, but inferior to the EPE-RM [11]. In our validation cohorts, the discrimination of ESUR alone was comparable with AUCs of 0.86–0.87. As a consequence, the RM combining MR including the ESUR classification and clinical parameters did not enhance the accuracy of EPE prediction in this validation cohort with specialist uro-radiologists and a standardized MR reading using the ESUR classification.

Thirdly, we emphasize that the discrimination of the EPE-RM on external validation was high with AUCs of 0.85–0.88, with similar performance as other nomograms published, most of which have AUCs of over 0.80. Differences between them can only be investigated through direct comparisons.

A strength of the present EPE-RM is the individualised risk assessment of EPE. This may be important when planning RP. With the information derived from the EPE-RM, a nerve-sparing approach can be planned in which, for example, an appropriate intraoperative frozen section examination is performed in cases with an increased risk in order to also reduce the rate of positive surgical margins [29, 30].

Our study has limitations. First, prevalence-dependence of the RM limits its generalizability. Transferability to other populations may be limited by the high prevalence of EPE in our subgroups [7, 8]. If the EPE-RM is applied to populations with lower prevalence, the predicted probabilities might be overestimated. Thus, in order to correctly determine the individuals` risk of harbouring EPE, it is mandatory to be aware of the EPE prevalence in the current population (43% in our cohort) to possibly adjust the RMs` intercept. An explanation for the high prevalence might be, that patient with advanced local disease have a higher likelihood of being referred to an academic tertiary referral center. In addition, the guideline-based recommendations for Active Surveillance (AS) are strictly followed in our centres. The EPE-RM has shown a benefit in the original work compared to the ESUR alone. This benefit could not be reproduced by our data suggesting to only apply the ESUR classification to predict EPE, rather than calculating the EPE-RM. One reason for the exceptionally good discriminatory ability of the ESUR classification in our study may be due to the fact that the mpMRIs were (re-)read by highly experienced uro-radiologists. This may have biased the discrimination rate. We also did not assess for interobserver variability for ESUR classification. Nevertheless, advanced risk modelling is complex and should be validated for the own patient population.

## Conclusions

On bicenter validation in cohorts at high risk of EPE, the EPE-RM had good discrimination and calibration to predict side-specific EPE. This provides benefit in the decision-making process for patient-tailored radical prostatectomy. However, when standardized ESUR scoring of mpMRI by expert radiologists is performed, the discrimination was comparable.
